# The AI Concierge: institutional design for recording, reconnection, and mutual growth at SPring-8-II

**DOI:** 10.1107/S1600577526008507

**Published:** 2026-09-14

**Authors:** Osami Sakata

**Affiliations:** a Japan Synchrotron Radiation Research Institute (JASRI) 1-1-1 Kouto, Sayo-cho Sayo-gun Hyogo679-5198 Japan; ESRF – The European Synchrotron, France

**Keywords:** synchrotron radiation facilities, knowledge infrastructure, tacit knowledge, institutional design, artificial intelligence

## Abstract

Synchrotron experiments generate not only data but also a continuous stream of tacit branching judgments: which sample to continue with, when to reprioritize batch groups, how to interpret ambiguous results. These judgments are rarely recorded, because the same documents that could capture them simultaneously serve as instruments of performance evaluation. This paper proposes a minimum operational specification (MVIS) for the AI Concierge, an institutional support layer comprising eight elements: capture pathway, AI-generated candidate draft, human confirmation, tier assignment, knowledge-steward review, consent and notice, provenance, and reconnection output. The structural prerequisite is the institutional separation of an accountability layer (proposals and reports) from a knowledge layer (decision branches, negative results, interpretive uncertainty). The MVIS is grounded in two worked examples drawn from actual beam-time branching episodes at BL13XU, with mandatory content-type labels distinguishing source-supported facts from illustrative content and unavailable data. A boundary comparison against well kept logbooks, electronic laboratory notebooks, metadata catalogues, FAIR infrastructure, and retrieval-augmented generation systems clarifies what the AI Concierge adds and what it does not claim to replace. Governance rules for role separation, re-identification risk management, and staff contribution evaluation—decoupled from protected-record content and count—are specified alongside the specification itself.

## Abstract 2

This Scientific Comment proposes the AI Concierge as an institutional design for recording and responsibly reusing tacit experimental judgments at synchrotron facilities. It specifies a minimum operational model that combines AI-assisted drafting with human confirmation, governance, provenance, and safeguards for knowledge reconnection.

## 1. Introduction

Synchrotron radiation facilities are entering a new phase in which low-emittance storage rings, higher brilliance, and larger data flows are redefining experimental practice. The ESRF Extremely Brilliant Source and the Advanced Photon Source Upgrade exemplify this international movement, positioning source upgrades not only as technical renewals but also as changes in the scale and character of experimental work (Dimper *et al.*, 2014 ▸; ESRF, 2020 ▸; APS, 2019 ▸). SPring-8-II should be understood within this context (Tanaka *et al.*, 2024 ▸; RIKEN, 2026 ▸). As experiments become faster, more data-intensive, and more consequential at branching points, the judgments made at the experimental front line become harder to reconstruct after the fact.

The value of a synchrotron radiation facility should not be measured solely by the papers and output metrics it eventually produces. Over the course of an experiment, tacit judgments continually accumulate that never appear in the published record: which sample to continue measuring, when to reprioritize batch groups, how to interpret results that do not match expectation. The term ‘tacit judgment’ is used here in a narrower sense than by Polanyi (1966 ▸, pp. 3–4): not all embodied expertise, but the specific, situated choices made during beam time that determine whether and how an experiment proceeds. The recording gap is infrastructural, not individual: as studies of knowledge infrastructure emphasize, what an institution makes recordable and retrievable shapes what can later count as usable knowledge (Star & Ruhleder, 1996 ▸; Bowker & Star, 1999 ▸).

The recording gap persists not from lack of tools but from a structural conflict: the documents in which branching judgments could be recorded simultaneously serve as instruments of performance evaluation. When the same report must demonstrate both that beam time was productive and that candid uncertainty was preserved, it is a natural institutional pressure—not a failure of integrity—to frame outcomes constructively. The remedy must therefore be structural rather than normative: the recording function and the evaluation function must be institutionally separated.

This paper proposes, as its central contribution, a *minimum operational specification* (MVIS) for the AI Concierge—a human-accountable institutional support layer that records, classifies, and reconnects experimental branching judgments. The specification comprises eight institutional elements (Section 4) together with the governance framework that makes the knowledge layer it populates credible and safe (Section 5). The novelty is not AI or retrieval technology as such, but the institutional composition: the structural separation of recording from evaluation, role-separated governance, and the specific design choices that distinguish this layer from existing infrastructure. That existing infrastructure comprises well kept logbooks, electronic laboratory notebooks (ELNs), metadata catalogues, FAIR data infrastructure, and retrieval-augmented generation (RAG) systems.

The design is grounded in two worked examples from the author’s own beam-time experience at BL13XU (Section 3), with mandatory content-type labels (source-supported [S], author recollection [R], proposed field [P], illustrative [I], unavailable [U]) applied throughout. Claims that depend on companion manuscripts currently submitted or in press are either restated within this paper or identified as out of scope; no central argument depends on inaccessible work.

This paper presents one author’s perspective on SPring-8-II institutional design; it does not describe a confirmed implementation plan or prejudge official decisions by JASRI or RIKEN. The broader challenge—tacit experimental judgment becoming harder to retain as facilities generate ever larger volumes of data—is not unique to SPring-8-II; the design logic developed here may offer one model for next-generation photon science facilities more broadly.

## 2. The recording problem at synchrotron endstations

### 2.1. Two patterns of knowledge loss

Scientific progress at synchrotron facilities depends not only on published data but also on the recombination of experiential knowledge. Two patterns explain why such knowledge is systematically lost.

*First pattern: disappearance of negative results.* When the same document must serve simultaneously as a knowledge record and an accountability instrument, there is natural pressure to frame outcomes in ways that emphasize constructive results. When a group discovers that a particular sample-preparation protocol fails under specific beamline conditions, or that an anticipated structural transition does not occur within the expected parameter range, that finding—genuinely valuable to the community—is lost. The next group repeats the same exploration from scratch, consuming beam time the community can ill afford to waste.

*Second pattern: interpretive drift.* A subtler knowledge loss occurs when interpretation gradually moves toward the outcome that was expected or desired. Data points at the boundary between noise and signal are quietly excluded. Parameters chosen opportunistically during analysis are later written up as if pre-planned. Decision branches considered but not pursued are replaced in the final report by a narrative that presents the observed results as the intended outcome all along. Each instance may seem trivial in isolation; cumulatively they introduce systematic bias into the field’s knowledge base—one that is difficult to detect because it is distributed across countless individually plausible reports.

Both pathologies share a structural root: the same documents serve as both knowledge records and evaluation instruments. It is this structural conflation—not any lack of integrity on the part of researchers—that makes complete and candid reporting difficult.

### 2.2. The accountability layer and the knowledge layer

The remedy must therefore be structural. An *accountability layer*—confirming that beam time was used as proposed and that results have been reported—should remain in place. Alongside it, a *knowledge layer* should be established in which experimental decision branches, negative results, and the gap between anticipated and observed outcomes can be recorded without feeding back into performance assessment.

This separation is analogous in principle to the no-fault incident reporting systems established in aviation and medicine, where decoupling reporting from punitive consequence is credited with improving the quality and completeness of safety-relevant information. The analogy is limited, however: in aviation and medicine, the performance evaluator is typically an external authority, not the immediate supervisor. In the endstation context, the beamline head commonly occupies both the supervisory and the evaluative role. The institutional design must therefore be explicit about who the knowledge steward is, how that role is separated from the evaluator’s function, and what access rules enforce that separation—questions addressed in Section 5.

**Figure 1:**
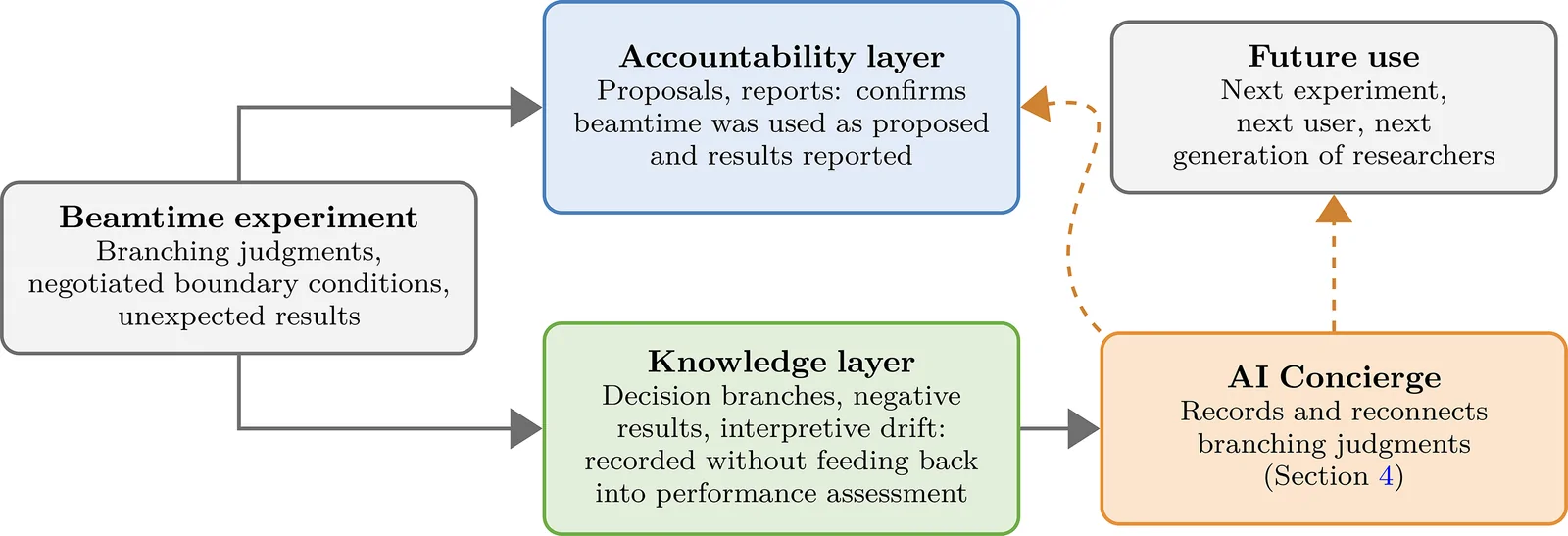
Institutional separation of the accountability layer and the knowledge layer. Tacit judgment recorded in the knowledge layer does not feed back into performance assessment. The AI Concierge (Section 4) populates the knowledge layer and reconnects its contents to future experiments and users.

Fig. 1 ▸ summarizes this separation and previews how the AI Concierge populates and reconnects the knowledge layer.

### 2.3. How the AI Concierge differs from existing infrastructure

The knowledge layer this paper proposes should not be conflated with existing recording systems. Table 1 ▸ sets out the boundary explicitly across six infrastructure types.

Three points require emphasis. First, ELNs *can* record rationale; the claim in this paper is not about capability but about institutional design (Kanza *et al.*, 2017 ▸). The recording gap persists with ELNs for exactly the same incentive reason it persists with paper notebooks: as long as records can be read by the performance evaluator, candid recording is suppressed. The AI Concierge addresses this not by replacing ELNs but by adding role-separated governance and an evaluation-firewall. Second, RAG systems over access-controlled corpora already exist (Lewis *et al.*, 2020 ▸). What is new here is not the retrieval technology but the institutional design. Three choices define it: what enters the corpus (human-confirmed records with provenance), how access is governed (tier-separated, evaluator-excluded), and how retrieval output is framed (case-based advisory prompts that make uncertainty explicit and require human judgment). Third, FAIR principles (Wilkinson *et al.*, 2016 ▸) and facility-level metadata catalogues—ICAT (Flannery *et al.*, 2009 ▸), SciCat (Pithan & Novelli, 2024 ▸), and the PaNOSC (PaNOSC Consortium, 2019 ▸) and ExPaNDS (ExPaNDS Consortium, 2019 ▸) community programmes—have substantially advanced the findability and reuse of experimental datasets. The AI Concierge is complementary: it operates upstream of formal metadata, at the point where judgment itself, rather than its eventual output, is exercised.

## 3. Beam-time branching episodes

What, concretely, should the knowledge layer record? The author provided hands-on user support for surface and interface X-ray diffraction experiments at the former BL13XU at SPring-8. BL13XU was decommissioned in March 2020 and its equipment discarded; no successor beamline has been planned. The question of whether the judgment cultivated there can be carried forward, even after the beamline’s physical existence has been lost, motivates the design proposed in this paper. Two recurring types of branching judgment illustrate the target.

**Branch 1 (sample substitution).** When diffraction measurements did not go well [R], it was necessary to determine whether the cause was X-ray-induced damage or a problem in the sample’s own fabrication. It was then necessary to judge whether to switch to a different sample within the same batch [S; see preceding sentence]. The *decision structure* is source-supported [S/R]: whether to continue with the current sample or switch to another sample within the same batch. The *actual decision outcome* is not recorded and cannot be reconstructed from the surviving documentation [U]. A Branch 1 decision—when it involves proprietary material composition or process history—is a natural candidate for Tier 4, entered into the knowledge base only with the user’s consent. This mapping is illustrative [I]; actual tier assignment is determined case by case.

**Branch 2 (batch reprioritization).** Samples typically comprised multiple batch groups, each assigned a priority; within limited beam time it was necessary to judge when to shift from lower priority to higher priority batch groups, or vice versa [S]. The *decision structure* is source-supported [S/R]: whether and when to reprioritize among batch groups under limited beam time. The *actual direction and outcome* are not recorded and cannot be reconstructed from the surviving documentation [U]. A Branch 2 decision often reflects facility-operational constraints rather than user-specific information, and is more naturally treated as Tier 2 (facility-internal) [I]. Again, actual tier is case by case.

A similar branching structure appeared at the former NIMS BL15XU, for HAXPES measurements. There, the judgment of how far to attenuate incident X-ray intensity for core-level versus valence-band spectra was made in real time and left no trace in the eventual report [R]. The recurrence across independent technical contexts—different facility, different method, same outcome—is what the absence of any recording mechanism, rather than the limits of individual memory, would predict.

At the time there was also a practice of noting, alongside experimental conditions, tendencies of user groups and points of caution as handover information for the next instance of user support. This handover information does not fit the format of a formal report, yet it shapes the quality of the support that follows. This is a second category of content the knowledge layer should accommodate.

The author cannot, at the time of writing, fully reconstruct the technical criteria used in making these branching judgments [R]. This is the recording gap as a lived instance, not a theoretical claim. The [U] fields in Tables 2 ▸ and 3 ▸ are the structural evidence of this gap; they are not omissions but findings. Had a recording mechanism such as the AI Concierge existed at the time, these judgments would have been traceable and reconnectable to future cases.

These episodes are not statistically representative. Their value lies in specifying what an AI Concierge would need to record in an actual SPring-8-II institutional context. They also demonstrate that technically consequential judgment can be indispensable during beam time yet leave almost no trace in the formal record.

## 4. Minimum operational specification of the AI Concierge

The AI Concierge denotes a human-accountable institutional support layer that records, classifies, and reconnects experimental branching judgments. It does not substitute for endstation staff judgment; it records, structures, checks, classifies, reconnects, and provides source-traceable provenance for that judgment. The eight elements below constitute the minimum operational specification.

### 4.1. Elements A–H

**A – capture pathway.** The AI Concierge extracts the minimum-necessary decision context from existing traces: logbook tags, instrument logs, preliminary spectra, and spoken exchange excerpts where consent exists. Capture follows data-minimization and purpose-limitation principles: only the information required to make the branching judgment traceable is drafted. Not every conversation or logbook entry generates a candidate record; capture is triggered when a decision point with knowledge-layer value is identified. Persistent storage of full conversation transcripts is not assumed. The data-minimization principle applies regardless of whether capture originates from dialogue, logbook tags, or instrument logs.

**B – AI-generated candidate draft.** The AI assembles a candidate record from the 16-field schema (Tables 2 ▸ and 3 ▸). This is a proposal, not a confirmation. A candidate record may include an AI-suggested rationale, clearly distinguished from a human-stated rationale. It becomes part of the confirmed record only after explicit human review and approval. If the AI-suggested rationale is not approved by a staff member, it is discarded; it does not enter the confirmed record.

**C – human review and confirmation.** The staff member reviews the draft and may modify, supplement, exclude individual fields, or discard the entire candidate record. Discard is always an option; no candidate record is confirmed without explicit human approval. The record becomes confirmed only after this step. Accountability attaches here, not at capture. The choice to discard carries no consequence. Capture follows data-minimization and purpose-limitation principles; only the decision context necessary for future understanding is retained.

**D – tier assignment.** The confirmed record is classified into one or more of four tiers by the staff member and knowledge steward, on a case-by-case basis. Tier 1: public information (published papers, technical documents). Tier 2: facility-internal information. Tier 3: anonymized case patterns for broader sharing. Tier 4: user-contributed information entered only with purpose-scoped consent. The tiers are independent classifications, not a derivation hierarchy; no tier is mechanically produced from another.

Tier 3 requires particular care: *Tier 3 is an independently governed classification, but not an origin-free record. It may be derived through human-reviewed abstraction from confirmed episode records, while retaining provenance to those source records. No automatic tier conversion occurs.* Before any Tier 3 record is confirmed, five steps are required: (i) staff scientific validity check; (ii) knowledge-steward confidentiality review; (iii) re-identification risk review; (iv) consent-boundary check for Tier 4 source material; (v) final knowledge-steward approval, logged.

**E – knowledge steward review.** The knowledge steward reviews confidentiality, contextual re-identification risk, tier eligibility, and consent scope independently of performance evaluation. *Anonymization and abstraction reduce risk but do not guarantee non-identifiability.* The knowledge steward assesses whether residual contextual re-identification risk is institutionally acceptable for the proposed reuse scope. If residual risk remains unacceptable, the record is not released as Tier 3; it remains under the source tier, is further abstracted, has its reuse scope narrowed, or is excluded from reuse. The steward is institutionally separated from the performance evaluator (see Section 5.1).

**F – consent and notice.** This element applies where capture involves user-contributed information or dialogue extraction. Four rules govern consent: (*a*) users receive prior notice of what may be captured and for what purpose; (*b*) consent is purpose-scoped, not a blanket permission; (*c*) users may stop capture at any time; (*d*) Tier 4 consent is revocable and is never a precondition for beam-time access. Refusal has no evaluative consequence.

**G – provenance and retention.** Each confirmed record retains a provenance link to its source traces (Moreau & Missier, 2013 ▸). For Tier 3 records, provenance links to the confirmed episode records from which the pattern was abstracted are retained internally (accessible to the knowledge steward for audit) but not exposed in the Tier 3 pattern itself. Retention is subject to periodic review rather than fixed expiry, because anonymization adequacy can change as related work is published elsewhere.

**H – reconnection output.** The AI Concierge returns case-based advisory prompts to future staff or users. Each prompt states: similarity basis, applicable conditions, residual uncertainties, access tier of the underlying record, and that final judgment belongs to the human. For Branch 1, a reconnection prompt would read: ‘*A prior case under partially similar conditions involved consideration of whether to continue with the current sample or switch to another sample within the same batch. The actual decision outcome and the criteria used at the time were not recorded. The present staff member must therefore determine whether the current conditions are sufficiently comparable and make a new human judgment.*’ The prompt reconnects to the decision structure, not to an executed outcome. Prompts do not issue commands or autonomous decisions.

**Figure 2:**
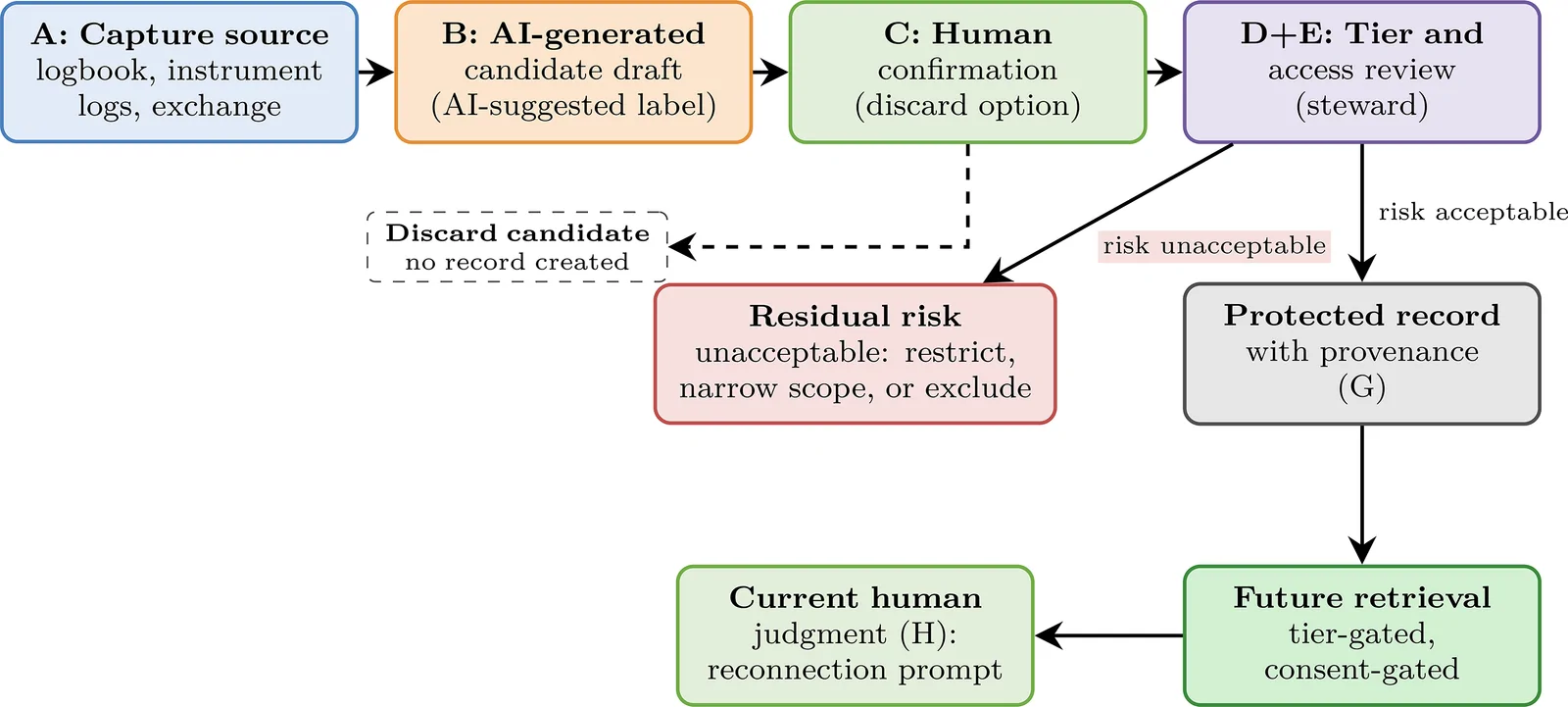
End-to-end workflow of the AI Concierge (elements A–H, Section 4.1). When the knowledge steward’s re-identification risk assessment (element E) finds residual risk unacceptable, the record remains under the source tier, is further abstracted, has its reuse scope narrowed, or is excluded from reuse. The discard pathway (element C) is always available: no candidate record enters the knowledge layer without explicit human approval.

Fig. 2 ▸ illustrates the end-to-end workflow including the residual re-identification risk pathway.

### 4.2. Record schema and worked examples

Tables 2 ▸ and 3 ▸ present worked records for Branch 1 and Branch 2, respectively, applying the 16-field schema with mandatory content-type labels. The [U] fields (unavailable—never recorded) are the structural evidence of the recording gap, not omissions. Illustrative [I] entries are explicitly labelled and must not be read as actual recorded criteria.

## 5. Governance, access, and safeguards

### 5.1. Role separation

The knowledge layer functions only when the roles that interact with it are institutionally separated. Tables 4 ▸ and 5 ▸ set out five roles, their permitted and prohibited actions, and access boundaries with conflict-of-interest mitigations. The most important boundary is between the knowledge steward and the performance evaluator.

*Performance evaluators have no routine access to protected knowledge-layer content for performance-evaluation purposes.* This is the critical operational constraint underwriting the accountability/knowledge separation. Without it, the knowledge layer collapses back into the accountability layer: staff who know that their records can reach the evaluator will record defensively or not at all.

The aviation and medicine analogy—no-fault incident reporting decoupled from punitive evaluation—is helpful as a motivating parallel but limited as a structural model. In those sectors, the evaluator is typically an external authority. In the endstation context, the performance evaluator is commonly the beamline head, who also has line authority over the staff whose records exist in the knowledge layer. The institutional design must therefore be explicit: access control denies evaluator routine knowledge-layer access; the audit log detects and records any exception use; policy states that record content and record count are not performance scores.

### 5.2. Re-identification risk and the Tier 3 pathway

Anonymization is necessary but not sufficient at a small endstation where the number of staff is limited and the context of decisions is often inferable even without names (Sweeney, 2002 ▸). A record can re-identify its author when the beamline, method, sample type, and time period are known to a knowledgeable reader—a situation typical of specialist beamlines where one or two staff members hold a specific technique.

The five-step Tier 3 review process (element D) exists precisely to prevent automatic conversion from episode records to Tier 3 patterns without this contextual re-identification assessment. *Anonymization and abstraction reduce risk but do not guarantee non-identifiability.* The knowledge steward assesses whether residual contextual re-identification risk is institutionally acceptable for the proposed reuse scope. If residual risk remains unacceptable, the record is not released as Tier 3: it remains under the source tier, is further abstracted, has its reuse scope narrowed, or is excluded from reuse. The Tier 3 pathway is therefore not a guaranteed route to broad sharing; it is conditionally available, subject to governance review.

### 5.3. Staff contribution evaluation

Two further mandated constraints protect the knowledge layer from perverse incentives. *Record content and record count are not performance metrics.* A system that allows evaluators to inspect record content, or to rank staff by the number of records confirmed, would create the precise Goodhart effect (Goodhart, 1975 ▸; Strathern, 1997 ▸) that makes measurement systems unreliable. The failure modes are familiar: defensive recording, volume over candour, and the suppression of genuinely informative but revealing entries.

Staff contributions in user support, knowledge stewardship, and method development are recognized through *portfolio evidence* that does not expose protected-record content to the evaluator. This evidence includes user feedback (aggregated and anonymized), peer observation, case sharing at annual reviews or beamline-operations meetings, and formal acknowledgement of steward function by a peer panel. Staff who develop new measurement methods, sample-environment solutions, or data-analysis approaches arising from endstation work are recognized through standard academic pathways (publications, patents, presentations) independently of the knowledge layer.

## 6. Institutional implications for SPring-8-II

SPring-8-II is designed as a major green upgrade combining a five-bend achromat lattice, a target emittance of 50 pm rad, and high-brilliance hard X-ray performance (Tanaka *et al.*, 2024 ▸). The transition intensifies rather than resolves the recording problem: experiments become faster, more data-intensive, and more consequence-laden at branching points; the judgments made during beam time become harder to reconstruct afterward. The institutional design proposed here should therefore be regarded as part of the facility’s knowledge infrastructure alongside its data and computing infrastructure.

The AI Concierge must be positioned as a *support for*, rather than a substitute for, endstation staff judgment. This is not a tone choice; it is a structural prerequisite. Suppose staff come to perceive themselves as a stage in a process by which their judgment is being replaced by AI. The motivation to record candidly is then eroded, the knowledge layer collapses, and the facility loses the tacit knowledge that the AI was meant to preserve. A substitution-oriented approach would ironically diminish the supply of tacit knowledge it seeks to substitute for. The AI Concierge proposed here is deliberately distinct from closed-loop autonomous experimentation (Kusne *et al.*, 2020 ▸; Pithan *et al.*, 2023 ▸; Toyama *et al.*, 2025 ▸), in which next measurement conditions are autonomously selected through active learning or Bayesian optimization. Closed-loop automation is best understood as a future extension that can be built on the knowledge base the AI Concierge accumulates, not as a replacement for the human-accountable layer itself.

Researchers responsible for experimental endstations grow as scientists through engagement with the scientific and industrial questions that users bring. This *mutual growth*—staff expertise sharpened through user interaction, users’ questions deepened through expert collaboration—is both a motivation for recording and a product of it. One example is the transfer of expertise developed in semiconductor heteroepitaxy to MOF thin-film research through user collaboration (Sakata & Ikemoto, 2025 ▸). Recording branching judgments creates a return visible to users: on-demand access to how similar decisions were handled in comparable past cases, within the limited beam time available. This gives users a direct stake in the mechanism, aligning incentives rather than relying on staff goodwill alone.

The facility-data-infrastructure layer and the judgment-recording layer are complementary. The APS Data Management System (Veseli *et al.*, 2018 ▸) demonstrates how experimental data, metadata, and provenance can be organized as a shared facility-wide resource; the AI Concierge builds on, rather than duplicates, such infrastructure. Over time, endstation staff might move from suppliers of tacit knowledge to originators of downstream science: surveying structural patterns across accumulated branching judgments, and forming new hypotheses from the recorded history of decision-making. Whether that shift happens depends on the knowledge layer being built on governance that staff trust. The design choices made at the outset, including role separation, evaluation firewalls, and re-identification safeguards, determine whether that future possibility becomes real.

The challenge is not unique to SPring-8-II. ESRF-EBS, APS-U, and comparable next-generation sources face the same structural problem: tacit judgment harder to retain as facilities generate ever larger data volumes (Dimper *et al.*, 2014 ▸; ESRF, 2020 ▸; APS, 2019 ▸). The current upgrade discourse centres on autonomous data infrastructure and FAIR principles; what this paper contributes additionally is attention to the human-centric knowledge circulation that autonomy-focused agendas do not by themselves address. The design logic developed here may offer one model, among others, for how facilities elsewhere might approach this challenge.

## 7. Limitations and conclusion

Several limitations require explicit acknowledgement. First, this is a minimum operational specification, not a confirmed implementation plan. The concrete granularity of recording, the specific technical interface for capture, and the governance arrangements best suited to SPring-8-II’s circumstances, are matters for facility-level design. Feasibility claims here are stated as design assumptions or technically plausible trajectories within SPring-8-II’s operational timeline, not as demonstrated capabilities.

Second, the branching episodes from BL13XU are analytically revealing examples, not statistically representative data. They illustrate what the AI Concierge would need to record; they do not constitute an evidence base for the recording-gap thesis more broadly. The recurrence of the same structural outcome at BL15XU—a different facility, a different measurement technique, the same result of judgment leaving no trace—is offered as additional evidence, not as systematic demonstration.

Third, the incentive obstacle—why endstation staff would invest time in populating any recording system—applies to the AI Concierge as much as to logbooks or ELNs. The design response (element C: discard option; portfolio recognition; reconnection return to users) addresses this partially but does not resolve it. A system that generates genuine reconnection value for staff and users is more likely to attract participation than one that does not. But whether the reconnection value materializes depends on a critical mass of records being accumulated, and that accumulation requires early adopter investment before the return is fully visible.

Fourth, the question of how to translate recorded tacit knowledge into evaluation metrics is deliberately unresolved. Any attempt to turn recording or mutual growth into metrics must confront Goodhart’s law (Goodhart, 1975 ▸; Strathern, 1997 ▸): when a measure becomes a target, it ceases to function as a good measure. The qualitative portfolio approaches described in Section 5.3 are proposed as a holding position. The OECD reference framework for scientific and socio-economic impact assessment (OECD, 2019 ▸) distinguishes impact assessment from narrower performance evaluation and may offer a useful conceptual basis; the concrete design is reserved for future work.

Fifth, the companion references cited in earlier versions of this paper (currently submitted or in press) are repositioned here: arguments that previously depended on them are either restated within this paper or identified as out of scope. No central claim in the current paper depends on inaccessible work. Full bibliographic details for companion papers will be supplied upon publication.

The contribution of this paper is the institutional composition rather than any individual component. The accountability/knowledge layer separation, previously articulated at a general level, is here given a minimum operational specification. That specification comprises eight elements with explicit design rationale, a 16-field record schema illustrated with Branch 1 and Branch 2 worked examples, a governance role table, a Tier 3 re-identification pathway, and a staff evaluation design decoupled from protected-record content and count. The specification is sufficient to make the mechanism checkable and to distinguish clearly what the AI Concierge adds from what existing infrastructure already supplies.

For SPring-8-II, treating the transition to a next-generation facility as an opportunity to redesign not only the data infrastructure but also the knowledge infrastructure is the starting point. The MVIS presented here is one concrete starting point for that redesign.

## 8. Declaration of generative AI and AI-assisted technologies in the writing process

Generative AI tools, including Claude and ChatGPT, were used during the preparation of this article to assist with idea development, language refinement, manuscript organization, consistency checking, and critical review. Their role was limited to supporting the author’s formulation, testing, and expression of the argument. They did not generate empirical data, perform independent analysis, make scholarly judgments, or determine the article’s conclusions. The author reviewed, selected, revised, and takes responsibility for all AI-assisted outputs, and is solely responsible for the article’s argument, content, accuracy, originality, and final wording. Consistent with prevailing authorship norms, these tools are not listed as authors.

## Figures and Tables

**Table 1:** Boundary comparison: what each infrastructure is designed to capture and where the AI Concierge differs ELN = electronic laboratory notebook; FAIR = Findable, Accessible, Interoperable, Reusable data principles; RAG = retrieval-augmented generation.

Infrastructure	Record creation	Cross-case retrieval	Role-separated governance	Reconnection to future judgment
Paper notebook	Manual, free-form	None	None	None
ELN	Digital, free-form; *capable of recording rationale*	Within-system search	None built in	None
Metadata catalogue (ICAT, SciCat)	Structured, post-experiment	Faceted search on metadata	None built in	None
FAIR infrastructure	Data objects and workflow metadata	Via persistent identifiers	None built in	None
Access-controlled RAG	Depends on corpus design	Semantic retrieval	Depends on deployment	Query-based; no structured uncertainty
AI Concierge	AI-drafted from traces; human-confirmed; S/R/P/I/U labels	Case-based; tier-gated; structured advisory	Role-separated; evaluator excluded from routine access	Structured prompts: similarity basis, conditions, residual uncertainty, human judgment required

**Table 2:** Worked record: Branch 1 (sample substitution, BL13XU) Content-type labels: [S] source-supported; [R] author recollection; [P] proposed field; [I] illustrative; [U] unavailable—never recorded. [U] fields are structural evidence of the recording gap, not omissions. [I] content is hypothetical. Table note: fields 7 and 9 are [U] because the human-stated rationale and the actual alternatives were not recorded and cannot be reconstructed. This applies to both Branch 1 and Branch 2.

#	Field	Branch 1: sample substitution (BL13XU)
1	Experiment ID	[S/P] BL13XU, surface/interface X-ray diffraction
2	Date/slot	[U: not retained in the surviving record]
3	User/group ID	[U] Tier 4 consent required; not available for archival BL13XU
4	Experimental objective	[U: specific objective not stated in the surviving documentation]
5	Sample/batch structure	[R/P] Multiple samples in batch; composition [U]
6	Triggering observation	[R] ‘Measurements did not go well’ [R]. [I: specific trigger criterion not recorded; intensity decline is illustrative, not the actual criterion]
7	Human-stated rationale	[U: criterion was not recorded; author cannot reconstruct]
8	AI-suggested rationale	[I] AI might illustratively suggest distinguishing beam-damage from fabrication failure. *Illustrative only; must be confirmed by staff before entering confirmed record; if not confirmed, discarded*
9	Alternatives considered	[U: actual alternatives not recorded]. [I: illustrative—beam damage versus fabrication failure versus alignment issue; not recorded criteria]
10	Decision made	Decision structure [S/R]: whether to continue with the current sample or switch to another sample within the same batch. Actual decision outcome [U]: not recorded and cannot be reconstructed from the surviving documentation
11	Evidence observed	[U: no specific measurement values recorded]
12	Residual uncertainty	[P: proposed field; uncertainty not formally recorded]
13	Tier candidate	[I: Tier 4 if proprietary sample information involved; Tier 2 otherwise. Illustrative—actual tier case-by-case; see element D]
14	Consent status	[P: Tier 4 consent required; not available for archival BL13XU]
15	Human confirmation	[P: required; not available for archival record]
16	Provenance links	[P: would link to instrument logs, logbook tags; not available for archival BL13XU]

**Table 3:** Worked record: Branch 2 (batch reprioritization, BL13XU) Same content-type label scheme as Table 2 ▸. [U] fields are structural evidence of the recording gap, not omissions. [I] content is hypothetical. Table note: fields 7 and 9 are [U] because the human-stated rationale and the actual alternatives were not recorded and cannot be reconstructed.

#	Field	Branch 2: batch reprioritization (BL13XU)
1	Experiment ID	[S/P] BL13XU, surface/interface X-ray diffraction
2	Date/slot	[U: not retained in the surviving record]
3	User/group ID	[S/P] Facility-internal (Tier 2); [U] user identity for Tier 4 portion
4	Experimental objective	[U: specific objective not stated in the surviving documentation]
5	Sample/batch structure	[R/P] Multiple batch groups, each with priority [R]; composition [U]
6	Triggering observation	[R/P] Approaching shift-decision point in limited beam time [R]. Remaining beam-time value [U]
7	Human-stated rationale	[U: criterion was not recorded; author cannot reconstruct]
8	AI-suggested rationale	[I] AI might illustratively list remaining beam time and change-over cost as relevant factors. *Illustrative only; requires human confirmation*
9	Alternatives considered	[U: actual alternatives not recorded]. [I: illustrative—continue current batch, switch to higher priority, partial allocation]
10	Decision made	Decision structure [S/R]: whether and when to reprioritize among batch groups under limited beam time. Actual direction and outcome [U]: not recorded and cannot be reconstructed from the surviving documentation
11	Evidence observed	[U: no specific operational data recorded]
12	Residual uncertainty	[P: proposed field]
13	Tier candidate	[I: Tier 2 for facility-operational factors; Tier 4 for user-specific priorities. Illustrative—actual tier case-by-case]
14	Consent status	[P: Tier 2 flag for facility-internal portion; Tier 4 consent for user-specific portion]
15	Human confirmation	[P: required; not available for archival record]
16	Provenance links	[P: same as Branch 1—would link to instrument logs, logbook tags; not available for archival BL13XU]

**Table 4:** Governance role separation (Part A): permitted and prohibited actions The knowledge steward and performance evaluator must be institutionally separated; where local separation is impossible, a cross-beamline or facility-level arrangement is required. See Table 5 ▸ for access boundaries and conflict mitigations.

Role	Permitted	Prohibited
Endstation staff	Review, edit, confirm AI-drafted records; provide rationale; set initial tier; discard candidate records at any stage	Finalize Tier 3 classification alone; alter another staff member’s confirmed record
Knowledge steward	Review confidentiality, re-identification risk, tier eligibility, consent scope; approve Tier 3 abstraction via five-step process; confirm provenance links; trigger periodic retention review	Perform performance evaluation; author scientific content; approve Tier 3 without completing all five steps
Performance evaluator (*e.g.* beamline head)	Evaluate staff via accountability-layer artefacts; recognize contribution via portfolio evidence (Section 5.3). Exception access only under defined conditions (see Table 5 ▸)	No routine access to knowledge-layer record content for performance-evaluation purposes. Must not use record content or record count as performance scores
System administrator	Manage access control, retention, audit logging, provenance links, backups	Evaluate scientific content; adjudicate tier; read content for non-operational purposes
User / data owner	Grant purpose-scoped Tier 4 consent; withdraw future-use consent prospectively; access own contributed material; request Tier confirmation	Access other users’ records; compel Tier changes for facility-internal (Tier 2) content

**Table 5:** Governance role separation (Part B): access boundaries and conflict mitigations Exception access for the performance evaluator applies only under research integrity, safety, legal, or serious incident investigation purposes and requires purpose limitation, explicit authorization, audit logging, and prohibition of secondary use.

Role	Access boundary and conflict mitigation
Endstation staff	Hard decoupling from evaluator; audit log of all confirmations
Knowledge steward	Where steward and evaluator must coincide locally, substitute a cross-beamline or facility-level review panel
Performance evaluator (*e.g.* beamline head)	Access control system denies routine knowledge-layer access; audit log detects and records any exception access; exception access requires: purpose limitation, explicit authorization, audit logging, prohibition of secondary use
System administrator	Least-privilege principle; separation of duties; immutable audit log; four-eyes on privileged access
User / data owner	Consent is purpose-scoped, revocable, never a precondition for beam time; refusal has no evaluative consequence

## Data Availability

No new data were generated or analyzed in support of this article.
